# Reasons for Preferring Somnoform to Nitrous Oxid as a General Anesthetic

**Published:** 1905-09-15

**Authors:** G. C. Bowles


### REASONS FOR PREFERRING SOMNOFORM TO NITROUS OXID AS A GENERAL ANESTHETIC.

BY G. C. BOWLES.

Read before the Michigan Dental Association, July 10, 1905.

Mark Twain, traveling in Europe, came across the bridge of Sighs. Standing upon that historic structure he sighed and remarked: "Alas, I shall never forgive Lord Byron for coming here before me. The fine things I would say about this place he has said. The emotions that shake my frame he has portrayed. The words that spring to my lips he has stolen and given them long since to the world. It is a plagiarism I cannot, I will not forgive."

In this I sympathize deeply, understandingly, with Mark, for on the subject of somnoform anesthesia, Dr. Rolland, of Bordeaux, has done for me what Lord Byron did for Twain. But I bear the Doctor no ill will.

Not only has he given us Somnoform, but he has written about it so ably, so understandingly, and from every phase of its use and action, that beside taking the words out of my mouth, he has apparently taken them out of the mouth of every writer whose remarks on the subject I have seen.

This being the case, I shall content myself with giving the reasons for my preference for somnoform after ten months experience, and not parade original quotations from Dr. Rolland's writings as samples of my own wisdom and understanding of the subject. No dentist would ever do such a thing, of course.

Therefore, for a concise and simple presentment of the subject, giving in condensed form the result of Dr. Rolland's investigations, the nature of somnoform, its mode of administration, its physiological effects, etc., let me at once refer you to the little treatise "General Anesthesia Produced by Somnoform," published by E. DeTray & Sons, to be had at the dental dealers for the asking. Herein will be found in permanent form more than I could say on the subject, did you care to listen. Let me say this, however, such of the statements and claims there made that the ordinary practitioner, administering the agent for practical and not experimental purposes, can verify, I have verified, and that thus far I am, in every way, highly pleased with the results.

In 1895, Dr Rolland undertook the teaching of the theory of anesthesia in the Bordeaux Dental College, and according to the introduction of the little treatise above mentioned, "he soon found himself confronted by the problem of how to produce a rapid anesthesia, easily brought about and presenting no danger; followed by a quick return to consciousness and having no ulterior and troublesome sensations." Doesn't that take the words out of your mouth? My practice does not demand very frequent use of a general anesthetic, but when it does it is for just that kind. My cumbersome old nitrous oxide outfit didn't fill the bill. A case presented last September, of which I shall speak again, forced me to do something. The result was that I put in a new, three-cylinder oxygen and nitrous oxide apparatus, and had the H. J. Caulkins Co. send for the first somnoform outfit brought to Detroit; probably the first brought to Michigan. Somnoform has practically solved the problem for me. I say "practically" for, taking my experience as a whole, it has not absolutely.

In the first place, I have had more than the average one percent of nausea cases, due chiefly to overdosing, the result of inexperience. One case was due to the administration of the anesthetic immediately after a full meal. In the second place, I narrowly escaped a thrashing from an infuriated traveling man who thought he was intoxicated, and that I was taking him home to his wife. He might have produced ulterior and troublesome sensations for me. However, I administer somnoform every time occasion requires a general anesthetic, while my new gas outfit stands beside the chair unused. This sufficiently indicates my choice.

Why do I prefer somnoform to nitrous oxide gas? Well, in the first place because, in my hands, it is more dependable. The patient can be assured unqualifiedly that he will know absolutely nothing whatever of the operation; indeed, that he will quite likely have some pleasant dream or enjoyable experience. The traveling man was happy until he thought his wife was likely to know about it. This assurance I could not give with gas. Occasionally I have found patients whom it was impossible to completely anesthetize with it, and frequently I have found those who, though seemingly unconscious, complained that they were not insensible to the pain and shock of the operation. From the frequent repitition of such remarks as, "Now, Doctor, give me enough, won't you? The last time I had a tooth pulled the dentist said he gave me gas enough to fill a balloon, but I felt it more than if I had taken nothing." I conclude that my experience is not peculiar to myself. Faulty apparatus, no doubt, has a good deal to do with the failure to produce complete narcosis with gas. The administration of too small an amount of the gas is another reason, and certainly in some cases the painful sensations complained of are purely subjective. The patient expects to be hurt, and the brain cells, acting upon the suggestion, probably at the first moment of returning consciousness, records the anticipated sensation. I had a demonstration of the subjectivity of such sensations about a year ago. This is the case referred to earlier in the paper.

The patient presented with the roots of three wisdom teeth to be extracted. The roots were loose and could have been almost painlessly removed without the use of any anesthetic, but the patient was not the kind to take chances; gas was administered, a root was snapped out in a twinkling, but even as it came the patient sat up, fully returned to consciousness. At the same sitting, three subsequent efforts were made to anesthetize the patient, but without avail.

Relating the experience to the demonstrator of an improved modern gas apparatus who was then exhibiting his outfit in the city, he said: "I will put that patient to sleep and keep her there an hour, if you wish it." I wished it. The patient, like Barkis, "was willin," and an appointment was made. During the course of an hour and a half, seven attempts were made to put that patient to sleep; gallons of gas were inhaled or forced down her throat under pressure, but she never completely lost consciousness for one moment. When the baffled demonstrator removed the inhaler, after the last effort, the very patient said, "You got it that time, didn't you?" I replied that I had not even tried. Said she, "Why, I felt the forceps slip off three times, then you tried again, and I felt it give way and come out." The root still being in place, she was easily convinced of the subjective nature of the sensations; but had the root fallen out, she would have known that three fruitless efforts had been made before it was finally removed.

With somnoform so far I have had no such experience. I have never had a patient complain of any unpleasantness such as the rough joltings and the shocks, that so frequently accompany the parting of the tooth from its environment under gas.

In the second place, I have nearly twice as long to operate. I rarely got more than thirty or forty seconds under gas. I easily get sixty or more under somnoform. This is a very great advantage, for it gives one time to go carefully and deliberately about the work. When extracting roots it is not necessary to make a grab and take gums, process and all; and one very rarely has to finish an operation with the patient struggling and screaming in the chair. If necessary, as with gas, somnoform may be administered two or three times in succession with the same ease and certainty as at first, and with little or no appreciable increase of discomfort to the patient.

In the third place, the absence of cyanosis saving onlooking friends from the fear of impending disaster, is no slight advantage. We may assure such friends that the ghastly pallor is entirely without significance, but the slightly improved color of the patient under somnoform is a more pleasant and convincing assurance to them, if not to ourselves than our most plausible affirmations.

Another marked advantage, especially in the case of children, and hysterically-inclined individuals, is that the period of excitement is so very brief that they relapse into a passive non-resistance almost before they have time to struggle. With three or four inhalations, excitement vanishes Three or four more and the gaze becomes fixed, and the flexed arm falls. Three or four more and a gentle snore announces that we may proceed with our operation.

Everyone is familiar with the great difference in the amount of gas required by different individuals. With somnoform, so far I have noted no such variations. I rarely give more than two or two and one-half cubic centimeters to children; nor more than four, and never more then five to adults. And usually there is enough left in the inhaler, after the operation is performed, to put me to sleep.

There are other advantages. For instance, the compactness of the apparatus, and the ease with which it is handled; the comparative ease with which it may be kept sterile; the simplicity of the parts and consequent lessened liability of derangement; the first cost and the cost to us.

These are all positive advantages from every point of view over gas. So far, as compared with gas, I have met with no disadvantages. Of course, in this country it yet remains to be seen whether it is as universally a safe agent as is nitrous oxide. If it proves so to be, it must certainly supplant that and every other at present employed agent for producing general anesthesia for all dental purposes.

As before stated, I did not know until I received a program a few days ago that I was expected to write about somnoform and how to use it. Thinking I was free to roam round the general subject of somnoform, I purposely omitted, it appears, the very thing I was expected to discuss. I had reasons for this. In the first place, I knew that printed matter giving this information and to which I had nothing original to offer would be on hand and free to all interested. In the second place, this information may be brought out in the discussion if thought necessary, and in the third place, I want to speak of a subject not assigned to me but which is entitled to consideration at a meeting so interested in the subject of anesthetics as is this. I refer to the analgesia, produced by rapid breathing, and first called to the attention of the profession by Dr. Bonwill, of Philadelphia, in 1880. Says Dr. Bonwill: "I can produce from rapidly breathing common air at the rate of one hundred respirations a minute a similar effect to that from ether, chloroform and nitrous oxide gas in their primary stages; and I can in this way render patients sufficiently insensible to acute pain from any operation where the time consumed is not over from twenty to thirty seconds."

Dr. Bonwill used this method to the exclusion of all others in his own practice, and Dr. Hewson, a surgeon of Philadelphia, employed it extensively and successfully in minor surgical operations. Why, then, has it not come into general use?

Because to the great majority of people it is extremely difficult to raise the respiration to one hundred and keep it there the required length of time. To many, it is a physical impossibility. Perhaps, too, experimenters failing to produce the analgesia Dr. Bonwill obtained, have not realized that they were actually, to a considerable degree, lessening the patients susceptibility to pain.

However this may, be for the past four years I have employed a modification of this agency and have found it an invaluable aid in the preparation of sensitive cavities, the removal of those troublesome nerve remnants, the handling of nervous children, etc.

I instruct the patient to breathe deeply and forcefully, at the rate of about forty respirations a minute. This focuses the mind on the part he has to play, and he is informed that according to the faithfulness with which he performs that part will be his freedom from pain. It also relieves the high nervous and muscular tension, that rigid attitude of expectancy, wherein the patient is ready instantly to react to the faintest intimation of pain, and he now stands almost without a wince what before he would not tolerate. The analgesic effect appears to be greater at the period of inhalation, and in particularly sensitive cases I say to the patient, "Now let us work together; you inhale deeply and vigorously, so that I can hear you, and I will cut at that time. If it hurts, don't stop, but breathe the harder."

The patients are often skeptical and want to know if it is Christian science or mind cure they are getting, but after trying it, all agree that it is a very great help, and some become highly enthusiastic in its praise.

There are several theories advanced to account for the effects produced. None of them are entirely satisfactory. I shall not burden you with recounting them. But I shall be pleased to demonstrate the value of this method when the operators get to work at the clinic Wednesday, if by chance any of them happens to have a sensitive patient. I do not claim that this method produces absolute insensibility to pain, but it very greatly reduces it, and makes the worst cases bearable. It is something that every practitioner can use many times every day. It requires no apparatus, no expense, leaves no bad after-effects and consumes no extra time. Try it. Work out its possibilities and you will find that it has its place along with somnoform, gas, pressure anesthesia and the other valuable aids toward the consummation, devoutly to be wished—Painless Dentistry.

### DISCUSSION.

Dr. A. L. LeGro: I consider somnoform a most wonderful anesthetic. About a year ago I purchased a somnoform outfit, while in St. Louis, and during the time that I have used it I have administered it 227 times. In all of those cases I failed to bring the patient to the inductive period but twice. One of these I am now convinced was due to a faulty inhaler with a small tubing, and the other I am at a loss to know the cause. I have had vomiting in one case. Patient about 65 years of age, an elderly lady, came to my office immediately following a very hearty meal. She had been suffering with valvular trouble for a number of years and her system was somewhat debilitated. I had very little trouble with her, however, about bringing her into the inductive stage of anesthesia.

My experience of one year with somnoform has naturally interested me in its physiological action. The one thing that has impressed me most about somnoform is the analgesic stage that follows the period of deep anesthesia. The analgesic stage in somnoform, I think, is more prolonged than with any other anesthetic. I have frequently operated for thirty seconds after the period of deep anesthesia, with no pain to the patient. Somnoform depresses the peripheral endings of the cardio inhibitory nerves, which causes a slight stimulation of the heart. Dr. Loeffler was recording the pulse of the patient I had to-day, and I think he noticed towards the latter part it came up a little. That seems to be the experience in all cases. Another very important thing about somnoform is the complete absence of restlessness. The patient seems to go into a quiet sleep and there is no change of color. The patient seems to be in a perfectly normal condition through the operation, and comes out of it as quietly as they pass into it. My experience has been that we have almost complete relaxation in about 85 percent of the cases, but I have noticed that in a number of cases, maybe 10 or 15 percent, there has been a marked muscular tension due, I suppose, to the subjectivity of the patient, as explained by Dr. Bowles. There is one physiological effect with this anesthesia I think that is characteristic of all general anesthetics and of some local, and that is a depression of the respiratory centers, but I think to a less marked degree than any agent I have thus far seen employed.

Dr. E. T. Loeffler: I do not think I could add anything in the way of an explanation as to the advantages of somnoform. I might say that our experience at the college has been very satisfactory; since we introduced the apparatus at the beginning of the year, we have used somnoform almost altogether. I do not mean by this that we have any prejudice at all against gas; our gas apparatus has been somewhat neglected, and yet that was not the real reason. It may be, perhaps, because a new thing works better than an old one, but the somnoform has proved very successful. We have taken pains to keep a record of all the cases, the time of induction, the duration, amount given and any peculiarity that may arise during the administration. It is almost too soon to speak dogmatically as I believe more ought to be done in the way of recording experiments. One may see it given any number of times, but it does not produce the same influence as when you personally administer it and carefully notice all the symptoms. We ought, in all cases, to investigate personally any agent that we are to use, so as to be in a position to judge of its efficiency. I had one case of valvular heart trouble, a case of mitral stenosis. The patient had been in the hospital for six months when I had occasion to give the anesthetic to him twice in one afternoon. He was suffering, besides, from some form of liver trouble, but there was a pronounced and characteristic heart lesion. The patient experienced no ill effects whatever from the somnoform, and we had occasion to watch him very carefully. I had two other cases in which there was a loss of a beat every five times, with substantially as good results. I do not feel that we should always fear patients that have heart troubles as much as some other conditions. I had two very unpleasant cases. In one case we found out very much more afterwards than we knew before; this was a lady who took tablets of acetanilid. Many women take headache powders, and they are all supposed to be harmless, but almost all contain acetanilid. This patient was anaemic and the symptoms which she manifested were somewhat alarming. The respiration seemd to be affected more than anything else; pupils were dilated and there was a numbness in the hands. A numbness of extremities indicates that there is a want of circulation; it may be only in some cases a stomach trouble, but is usually due to an anaemic condition. Another case in which we had trouble was a patient who was suffering from an ulcer of the stomach. She was affected much the same as the first one. Cases in which the patients are suffering from stomach trouble, or are addicted to the habit of taking headache powders, the somnoform should be used carefully. General practitioners will not, as a rule, give an anesthetic without knowing something about the patient's physical condition and preparing them beforehand. With healthy patients there is little risk with either gas or somnoform, but when we have a delicate patient, it would be well to learn all we can about the patient's condition. We are always, as Dr. Straith says, taking the life of our patients in our hands, and no matter how safe it may be, we should give the patient every possible chance in the way of preparation.

Dr. S. Straith: I want to say that since I expect to make my bread and butter from extracting, it is to my advantage to use the best thing for the purpose. While I am now a user of gas and of nothing else, I want to be ready when the proper time comes to adopt any other method that I believe is just as safe for my patients upon whom I operate and will give me the results. I think Dr. Loeffler has touched the keynote on at least one part of this discussion and that is, that experimenting should be done with the new things that come before us, especially such powerful agents for harm as general anesthetics. I think we are all prone to pick up anything that comes out which has a little money back of it to advertise it, and to launch it out on the people without sufficient knowledge. It is unsafe for us to use anything that we have not a good knowledge of, or that does not come well recommended. When we get our diploma we are given the privilege of administering any anesthetic that is on the market. However, we should be cautious. One of the things which count so much in favor of somnoform makes me afraid of it; that is the rapidity with which we get anesthesia. We get anesthesia much sooner than with gas. It is much more powerful and perhaps more dangerous. If I am properly informed, the principal substances from which we get somnoform are ether chlorides. Just a few days ago we had a death in one of our hospitals from chloroform, and a few days before that a death from ether. If it is derived from ether products, then we must have a dangerous anesthetic.

Dr. G. C. Bowles: Dr. Le Gro spoke of the normal condition of the patient under the administration of somnoform. In my own practice I have very little disturbance; the patients take it quietly and easily, but this afternoon in the clinic there was more disturbance than I ever had in my practice. I attributed it to the nervousness produced by the crowds and possible nervousness on the part of the administrator. The cases that Dr. Loeffler speaks of those who have stomach trouble and some heart trouble combined, are similar to the two cases that I had Saturday morning; one was the worst case that I ever had and the only one that alarmed me at all. The patient I have known for some time, and she has been suffering from catarrh of the stomach. I did not know, however, that she had any other serious trouble. I administrated the somnoform and the respirations were very shallow. It took me three times longer than it ordinarily does and ten times as long to return her to consciousness. When she did regain consciousness the respiration was difficult, the heart beat fell and for some time she could not see; when I spoke to her she made an effort apparently to talk and could not do so. I gave her brandy and aromatic spirits of ammonia and other remedies, and finally she revived, and then she told me that she was subject to attacks of that kind. She said that on another occasion she was taken with just such a spell on a street car and was carried into a physician's office; so this was an extremely bad case. We are experimenting, perhaps in this country, but this preparation has been used in Europe and in some of the hospitals in England exclusively since 1895, and it has been demonstrated there conclusively that it is a safe agent to use. It has been used here only a short time, comparatively, but it is not an experiment. The rapidity with which it produces anesthesia is due to its extreme volatility. It increases the circulation and brings about anesthesia in that manner.

